# Achievable rate as affected by active elements distribution in reconfigurable intelligent surfaces for wireless communication

**DOI:** 10.7717/peerj-cs.1207

**Published:** 2023-01-12

**Authors:** Dongming Li, Sixu Li, Guilu Wu, Chunxi Zhao

**Affiliations:** 1 College of Information Technology, Jilin Agricultural University, Changchun, China; 2 State Key Laboratory of Networking and Switching Technology, Beijing University of Posts and Telecommunications, Beijing, China; 3 School of Information and Communication Engineering, Hainan University, Haikou, China; 4 Information Center, Jilin Agricultural University, Changchun, China

**Keywords:** Reconfigurable intelligent surfaces, Achievable rate, Active elements distribution

## Abstract

To increase constantly the achievable rate of reconfigurable intelligent surfaces (RISs)-assisted communication systems, the traditional approaches are to deploy a few active elements randomly on the passive RISs. However, the effect of the geometry distribution of the deployment of the active elements is ignored in performance analysis. In this article, three types of geometry distribution with active elements on RISs, denoted as random distribution, uniform distribution, and eight-queens distribution, are discussed to analyze the affect on achievable rate in RISs-assisted wireless communications. Specifically, the optimal achievable rate is obtained according to the predefined codebook, and the codebook is determined by the reflection beamforming codeword related to the active elements geometry distribution in RISs. Simulation results show that different geometry distribution of active elements in RISs causes different influences to achievable rates. The eight-queens distribution proposed in this article for active elements in RISs brings the highest achievable rate compared with random distribution and uniform distribution. In the passive RISs surface, the distribution of a few active elements is limited by the eight-queens, further enhancing the achievable rate of the wireless communication system. This method has a 7% improvement over the conventional method.

## Introduction

The network capacity of the sixth-generation communication system is expected to grow a thousandfold. Ubiquitous wireless connectivity will become commonplace. However, highly complex networks, high-cost hardware and increasing energy consumption have become the key issues facing wireless communications in the future. Among the candidates for the new technology, reconfigurable intelligent surfaces (RISs) stands out for its unique low-cost, low-energy, programmable, and easy-to-deploy features (Huang et al., 2019; Yuan et al., 2021; ElMossallamy et al., 2020). It is a surface composed of a large number of passive elements made of electromagnetic materials (EM) (Wu et al., 2021; Basar et al., 2019; Pan et al., 2021; Yan, Yuan & Kuai, 2020). Each of the RISs’ small reflective units can be edited to set the reflection parameters, thus changing the original propagation path of the emitted signal. This results in signal enhancement and path loss reduction. The characteristics, applications, and prospects of RISs have attracted extensive research interest. In particular, increasing the achievable rate of RISs-assisted communication systems has been challenging work (Jung et al., 2020, 2021; Perovic et al., 2021). The difference between RISs and conventional relaying techniques is that radio frequency (RF) chains and amplifiers are not required. Thus power consumption and hardware costs are largely reduced.

Since programmable metamaterials were proposed by Prof. Tiejun Cui’s research team, research on RISs has been extremely extensive (Basar, 2019). Examples include data rate analysis for RISs-assisted communication (Hu, Rusek & Edfors, 2017a, 2017b, 2018), power optimization (Yu, Xu & Schober, 2019; Jung, Saad & Kong, 2021), channel estimation (Nadeem et al., 2020; Taha, Alrabeiah & Alkhateeb, 2021), deep learning-based communication design (Liaskos et al., 2019; Taha, Alrabeiah & Alkhateeb, 2021) and reliability analysis (Jung et al., 2019), *etc*. In particular, the study of achievable rates for RISs-assisted communication has received increasing attention in recent years. In order to enhance the achievable rate of RISs-assisted communication systems, some studies have started to focus on the structure of RISs elements. The element-grouping strategy was adopted in Zhang et al. (2021), where each element in one group utilized the same reflection coefficient. Two deep learning-based networks were then designed and concatenated to obtain channel knowledge for improving the achievable rate. In order to reduce the training overhead, the accuracy of channel knowledge was not ideal. Taha, Alrabeiah & Alkhateeb (2021) proposed that a small number of active elements were distributed on the surface of passive RISs to collect channel knowledge. The collected channel knowledge was trained with deep learning model. It not only reduced the computational complexity but had a higher achievement rate than the traditional method. On the basis of the structure of RISs in Taha, Alrabeiah & Alkhateeb (2021), Aygül, Nazzal & Arslan (2021) optimized the model by using the correlation existing in the previous sampling channel. However, the influence of the distribution structure of active elements in the RISs-assisted communication system was ignored in the models designed by Taha, Alrabeiah & Alkhateeb (2021) and Aygül, Nazzal & Arslan (2021). In the prior work, the distribution of active elements was random, and random distributions can lead to extreme cases. For example, all active elements are distributed in the same corner or line. This will affect the mapping of acquisition channel knowledge and the final result. In this article, we propose a new distribution method: the eight-queens distribution. At the same time, the traditional random distribution and the uniform distribution in one of the extreme cases were used as the control experiment to discuss the effect of active elements distribution on the achievable rate for RISs-assisted communication systems. The process of building the simulation experimental model was referred to the literature (Alam et al., 2022; Talukder et al., 2022; Khan et al., 2022). The simulation results show that the distribution of eight-queens is the closest to the upper limit among these methods and is 7% higher than the traditional methods. This article is a theoretical study based on the assumption of perfect channel knowledge. Due to the realistic conditions, it is difficult to achieve perfect channel knowledge in real physical environments.

In this article, the research contributions in the research related to RISs-assisted wireless communication systems are as follows:

1) The effect of the distribution structure of the active elements on the RISs on the achievable rate of wireless communication is analyzed.
2) The distribution structures of three types of active elements, random distribution, uniform distribution, and eight-queens distribution, were constructed.
3) For the novel eight-queens distribution method, the control is implemented with uniform distribution and traditional random distribution, respectively. The experiments prove that the distribution method of the active elements has some influence on the results, and verify that the proposed eight-queens distribution method in this article is the best.

The main structure of the rest of the article is as follows. “System Model” constructs a wireless communication system assisted by RISs. “Problem Formulation” expresses the mathematical expression of the problem in this article. “Analysis of Active Elements Distribution and Effect on Achievable Rate” analyzes the reasons for the effect of active elements distribution on the achievable rate and describes three types of distribution. “Numerical Results” describes the experimental setting and the experimental procedure. And the experimental results were analyzed and discussed. “Conclusion” summarizes the full article and provides an outlook for future work.

*Notation:* In this article, boldface uppercase letters, boldface lower case, and lower/upper case represent matrices, vectors, and scalars, respectively. For a matrix **A**, its transpose and conjugate are denoted ${{\bf A}^T}$ and ${{\bf A}^ * }$, respectively. ${\rm {\mathbb E}}[ \cdot ]$ denotes expectation. ${\rm \Vert }{\bf A}{\rm \Vert }$ is the determinant of **A**. ${\bf A} \odot {\bf B}$ is the Hadamard product of **A** and **B**. ${\rm {\cal N}}(m,R)$ is a complex Gaussian random vector with mean $m$ and covariance *R*.

## System model

### Network model

**Figure 1:**
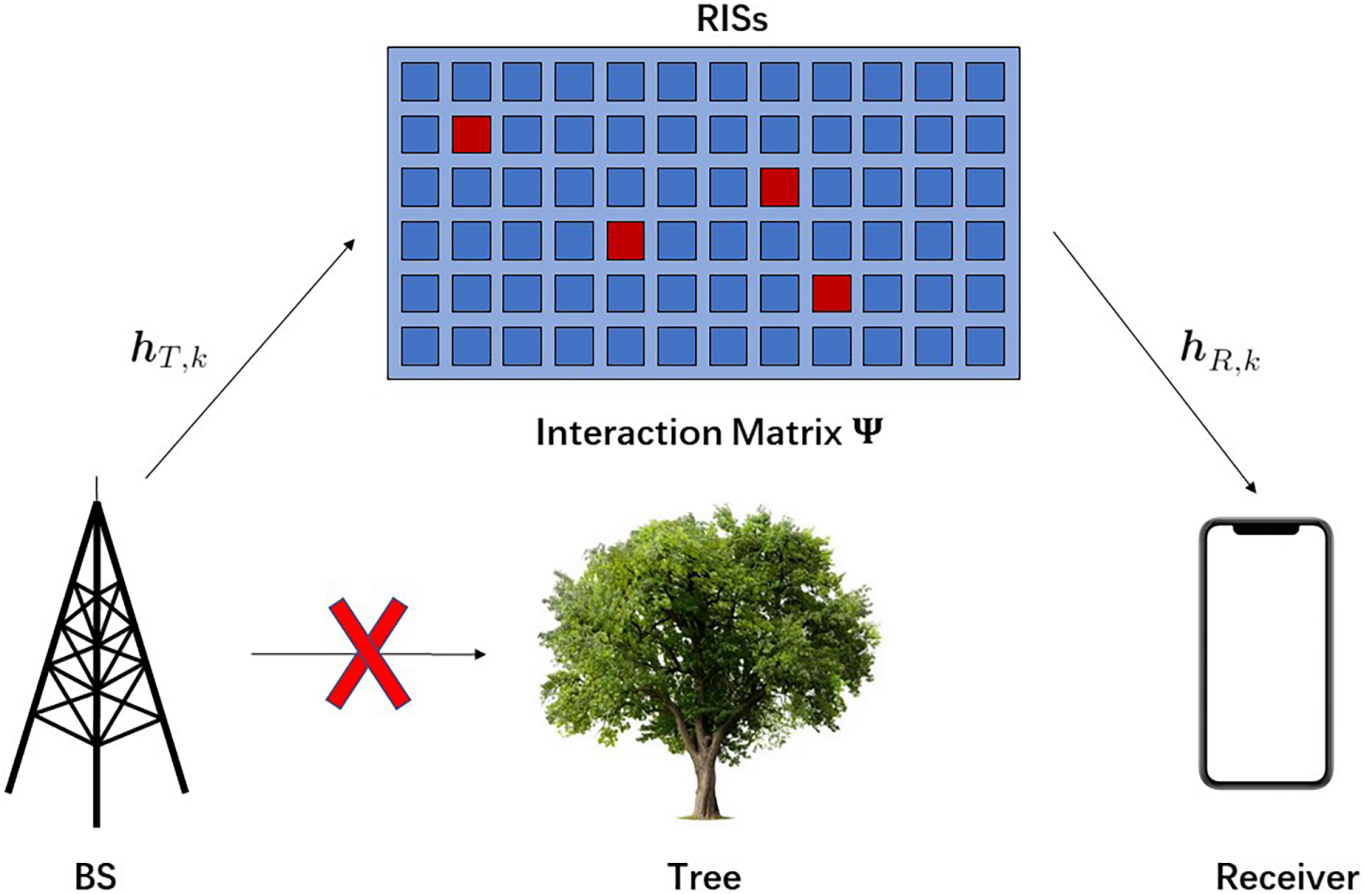
The system model of an RISs-assisted transceiver system.

Considering an RISs-assisted communication system which is consisted of base station (BS) and Receiver with one signal antenna, and RISs with *M* reflecting elements, as shown in Fig. 1. The direct link between BS and Receiver is blocked. An OFDM-based *K* subcarrier system is adopted, and ${h_{T,k}},{h_{R,k}} \in {{\mathbb C}^{M \times 1}}$ are the $M \times 1$ channels from BS to RISs, and RISs to Receiver at the $k$th subcarrier (Zheng & Zhang, 2019; Yang et al., 2020). Thus the received signal at the Receiver can be written as

(1) $$\matrix{ {{y_k}} \hfill & { = h_{R,k}^T{\Psi _k}{h_{T,k}}{s_k} + {n_k},} \hfill \cr }$$

(2) $$\mathop = \limits^{(a)} {({h_{R,k}} \odot {h_{T,k}})^T}{\psi _k}{s_k} + {n_k},$$

where ${s_k}$ represents the transmitted signal over the $k$th subcarrier, and is constrained by ${\rm {\mathbb E}}[|{s_k}{|^2}] = \displaystyle{{{P_T}} \over K}$, with ${P_T}$ denoting the total transmit power, and ${n_k}\sim{{\rm {\cal N}}_{\mathbb C}}(0,\sigma _n^2)$ is the receive noise. The matrix ${\Psi _k}$, that is called the RISs interaction matrix, represents the interaction of the RISs with the incident signal from the BS. The unique goal is to achieve the achievable rate of the system by adjusting the RISs (matrix ${\Psi _k}$). It is noted that ${\Psi _k}$ is a diagonal matrix here, and it can be denoted as ${\Psi _k} = diag({\psi _k})$. The diagonal matrix ${\psi _k} = {\beta _1}{\alpha _1}\exp (j{\theta _1}),...,{\beta _M}{\alpha _M}\exp (j{\theta _M})$, where ${\beta _m} \in \{ 0,1\}$, 1 represents passive elements and 0 represents active elements, $m \in \{ 1,2,...,M\}$, ${\alpha _m} \in [0,1]$ represents the amplitude coefficient, and ${\theta _m} \in [0,2\pi )$ represents phase shift of RISs. For simplicity, two assumptions are made on these interaction vectors. First, the RISs element uses only the phase shift controller to adjust the phase shift. Second, all subcarriers will be given the identical phase shift, *i.e*., ${\psi _k} = \psi ,\forall k$. Further, the interaction vector $\psi$ is called the reflection beamforming vector.

### Channel model

For channels ${h_{T,k}}$ and ${h_{R,k}}$, the broadband geometric channel model is used (Taha et al., 2020). A BS-RISs uplink channel ${h_{T,k}}$ and an RISs-Receiver uplink channel ${h_{R,k}}$ consisting of *L* clusters. And each cluster $\ell$ contributes with one ray from the transmitter to the RISs. Each ray of time delay ${\tau _\ell } \in {\mathbb R},{\theta _\ell },{\phi _\ell } \in [0,2\pi )$ represents azimuth/elevation angles of arrival, and a complex coefficient ${\alpha _\ell } \in {\mathbb C}$. ${\rho _T}$ represents the path loss between the BS and the RISs, and $p(\tau )$ characterizes the pulse shaping function for ${T_S}$-spaced signaling evaluated at $\tau$ seconds. The delay- $d$ channel vector ${h_{T,d}}$ between the BS and the RISs can be written as

(3) $${h_{T,d}} = \sqrt {\displaystyle{M \over {{\rho _T}}}} \sum\limits_{\ell = 1}^L {{\alpha _\ell }} p(d{T_S} - {\tau _\ell }){\rm \bf a}({\theta _\ell },{\phi _\ell }),$$

where ${\bf a}({\theta _\ell },{\phi _\ell }) \in {{\mathbb C}^{M \times 1}}$ represents the array response vector of the RISs. Base on the delay- $d$ channel, the frequency domain channel vector at subcarrier $k$, ${h_{T,k}}$ can be written as

(4) $${h_{T,k}} = \sum\limits_{d = 0}^{D - 1} {{h_{T,d}}} {e^{ - j\displaystyle{{2\pi k} \over K}d}}.$$

Considering a channel model of block-fading, ${h_{T,k}}$ and ${h_{R,k}}$ are considered to stay constant within the channel coherence time, ${T_C}$, which is strongly linked to the dynamics of the environment and the user mobility.

## Problem formulation

As mentioned earlier, our main target is to optimize the achievable rate of the receiver by designing the RISs reflection beamforming vector $\psi$. According to the network model and channel model described in in the previous section, the achievable rate *R* can be defined as

(5) $$\matrix{ R \hfill & { = \displaystyle{1 \over K}\sum\limits_{k = 1}^K {{{\log }_2}} (1 + SNR|h_{R,k}^T\Psi {h_{T,k}}{|^2}),} \hfill \cr }$$

(6) $$= \displaystyle{1 \over K}\sum\limits_{k = 1}^K {{{\log }_2}} (1 + SNR|{({h_{T,k}} \odot {h_{R,k}})^T}\psi {|^2}),$$

where $SNR = \displaystyle{{{P_T}} \over {K\sigma _n^2}}$ represents the signal-to-noise ratio. As we all know, the reflection beamforming vector is controlled by a phase shifter. However, it is assumed that the RISs elements take only one angle from the set of discrete quantized angles due to hardware restrictions. Therefore, the reflection beamforming vector $\psi$ is assumed that can only be selected from the predefined codebook ${\rm {\scr{P}}}$. It is assumed that each candidate reflection beamforming codeword in ${\rm {\scr{P}}}$ is implemented using a quantized phase shifter. Based on this assumption, the main goal of this article is to seek the optimized reflection beamforming vector ${\psi ^ * }$ constrains by the following formula

(7) $${\psi ^ * } = \mathop {{\rm argmax}}\limits_{\psi \in {\rm {\scr{P}}}} \sum\limits_{k = 1}^K {{{\log }_2}} (1 + SNR|{({h_{T,k}} \odot {h_{R,k}})^T}\psi {|^2}).$$

The formula above will conclude the optimized achievable rate ${R^ * }$, and it can be written as

(8) $${R^ * } = \mathop {{\rm max}}\limits_{\psi \in {\rm {\scr{P}}}} \displaystyle{1 \over K}\sum\limits_{k = 1}^K {{{\log }_2}} (1 + SNR|{({h_{T,k}} \odot {h_{R,k}})^T}{\psi ^ * }{|^2}).$$

Because of the limitations of using one interaction vector $\psi$ for all subcarriers, the optimization problem in Eq. (8) does not have a closed form solution. Owing to the discrete phase shifts are restricted in a limited set ${\rm {\scr{P}}}$, the exhaustive search on the codebook ${\rm {\scr{P}}}$ can result in a large cost of the optimal solution. In Taha, Alrabeiah & Alkhateeb (2021), the author proposed a deep learning method, multi-layer perceptron (MLP), to solve the problem of the high complexity of the exhaustive search. Based on Taha, Alrabeiah & Alkhateeb (2021), the effect of the distribution of the active elements on the achievable rate will continue to be discussed.

## Analysis of active elements distribution and effect on achievable rate

### Effect of active elements distribution on achievable rate

The random distribution of active elements on the RISs is uncertain and prone to extremes. For example, active elements are distributed in the same line or clustered together. As a result, the mapping of channel knowledge collected by active elements to passive elements may be influenced, further affecting the achievable rate of the communication system. To verify this possibility, the simulations are performed adopting the random distribution, the uniform distribution on the same line, and the eight-queens distribution, respectively. Through simulations, the assumptions proposed are proved that restricting the distribution of active elements can increase the achievable rate of the communication system.

### Active elements distribution expression

#### Random distribution

According to the above introduction, the value of ${\beta _m}$ can represent the distribution structure of active elements on the RISs. Furthermore, when $\forall {\beta _{{i_c}}} = 0,i \in 1,2,...,M,c \in 1,2...,8$, it is called as the random distribution.

#### Uniform distribution on the same line

There is a special case in the distribution structure of active elements. The active elements are distributed in the same column on the RISs, and the spacing of each active element is equal. Then the distribution is called as a uniform distribution on the same line. It can be expressed as

(9) $${\rm }\forall {\beta _{\big(\textstyle{M \over 8} - b\big) + \textstyle{M \over 8} \times (c - 1)}} = 0,b \in \{ 1,2,...,\sqrt M \} ,c \in \{ 1,2,...,8\} .{\rm }$$

#### Eight-queens distribution

The eight-queens problem was proposed by a chess player Max Bethel in 1848. It is a typical case of a backtracking algorithm. The problem is expressed as eight-queens are placed on the $8 \times 8$-grid chess. Eight-queens can’t attack each other, that is, any two queens can’t be placed in the same row, column or slash. Therefore, we will utilize the characteristics of eight-queens problem to limit the distribution of active elements. Let $A{1_{row}} = {\beta _{{i_{{c_1}}}}}/\sqrt M$, $A{2_{row}} = {\beta _{{i_{{c_2}}}}}/\sqrt M$, $A{3_{row}} = {\beta _{{i_{{c_3}}}}}/\sqrt M$, $A{4_{row}} = {\beta _{{i_{{c_4}}}}}/\sqrt M$, $A{1_{col}} = {\beta _{{i_{{c_1}}}}}\% \sqrt M$, $A{2_{col}} = {\beta _{{i_{{c_2}}}}}\% \sqrt M$, $A{3_{col}} = {\beta _{{i_{{c_3}}}}}\% \sqrt M$, $A{4_{col}} = {\beta _{{i_{{c_4}}}}}\% \sqrt M$, ${c_1},{c_2},{c_3},{c_4} \in \{ 1,2,...,8\} ,{c_1} \ne {c_2},{c_3} \ne {c_4}$. The symbols ‘/’ and ‘%’ denote rounding and remainder, respectively. Let $\forall {\beta _{{i_c}}}$ follow the following conditions

(10) $$\forall A{1_{row}} \ne \forall A{2_{row}},$$

(11) $$\forall A{1_{col}} \ne \forall A{2_{col}},$$

(12) $$\forall |\forall [A{1_{row}} - A{2_{row}}]| \ne \forall |\forall [A{3_{row}} - A{4_{row}}]|,$$

(13) $$\forall |\forall [A{1_{col}} - A{2_{col}}]| \ne \forall |\forall [A{3_{col}} - A{4_{col}}]|,$$

where Eq. (10) restricts that the active elements are not in the same row, Eq. (11) restricts that the active elements are not in the same column, Eqs. (12) and (13) restrict that the active elements are not in the same slash. This distribution is called as the eight-queens distribution.

## Numerical results

### Simulation description

The data set adopted is the DeepMIMO, which is a public data set (Alkhateeb, 2019). BS3 in the ‘O1’ scene in the DeepMIMO dataset is selected as the RISs. The antenna units on BS3 are regarded as RISs units. In order to better simulate the effect of environmental on the real channel, ray tracing is utilized to catch the dependencies of critical environmental factors. Therefore, scenario ${\rm O1}\_{\rm 28}$ is chosen to simulate actual physical environment. The simulation is based on the deep learning model MLP (Taha, Alrabeiah & Alkhateeb, 2021). The tool used in the simulation experiment is Matlab R2019b. The operating system is Linux and the system version is Ubuntu 16.04 LTS.

### Parameter setting

**Figure 2:**
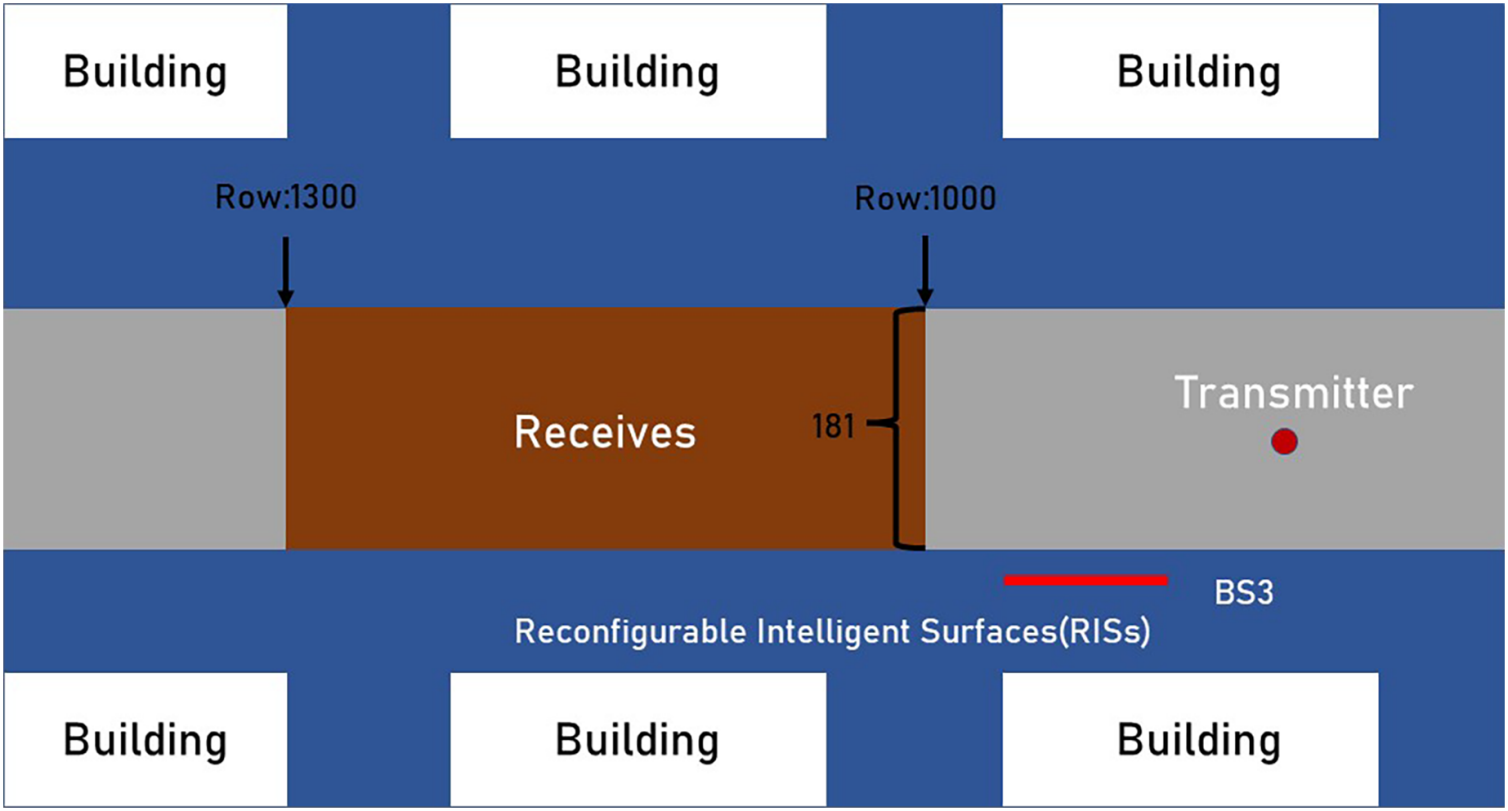
The system model of an RISs-assisted transceiver system.

In Fig. 2, BS3 in ‘O1’ scenario is considered as RISs, and fix the transmitter in row 850 and column 90. In the ‘O1’ scenario, the location of the receiver in the candidate area includes rows R1000 to R1300 and each row includes 181 points, a total of 54,300 points. In addition to special instructions, the RISs used in the simulation adopts 32 × 32 (M = 1,024) antennas uniform planar array (UPA) at mmWave 28 GHz setup. The gain of the antennas elements are set to 3 dB and the transmission power is set to 35 dbm. The selection of active elements in the UPA antennas follows the description in “Analysis of Active Elements Distribution and Effect on Achiev-Able Rate”. In Table 1, additional parameters of a DeepMIMO dataset are summarized.

**Table 1:** The adopted DeepMIMO dataset configuration parameters.

Parameter	Value
Number of RIS antennas	$({M_x},{M_y},{M_z}) = (1,8,8);(1,16,16);(1,24,24);(1,32,32);$
Antenna spacing	0.5
System bandwidth	100 MHz
Number of OFDM subcarriers	512
OFDM sampling factor	1
OFDM limit	64
Number of paths	1,5

### Simulation results

**Figure 3:**
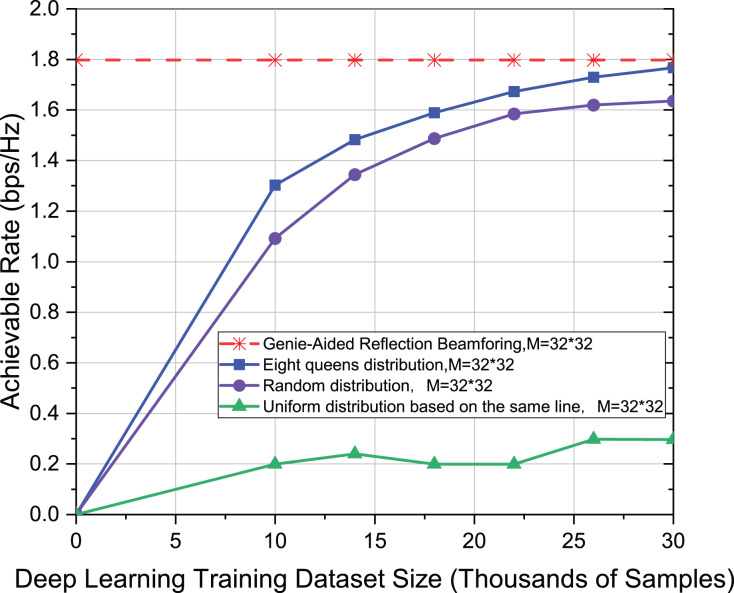
Comparison of achievable rate of eight-queens distribution, random distribution and uniform distribution on the same line when M = 32 * 32.

Firstly, the effect of different distribution of active elements on the achievable rate is studied in the deep learning model for different data set sizes. Secondly, the size of the reflective surface is $M = 32 \times 32$ and the number of active elements is Ma = 8. The experiment uses the mmWave 28 GHz scenario and the channel with only one path, *i.e*., L = 1. The model and parameter settings used in this experiment are the same as Taha, Alrabeiah & Alkhateeb (2021). In Fig. 3, however, since the distribution of active elements is limited by the eight-queens, the result is better than random distribution, and improves the achievable rate to be 7%. The reason is that the eight-queens distribution limits the distribution structure of the active elements. The eight-queens distribution ensures that the active elements are relatively dispersed. In contrast, the effect is the worst when the active elements are uniform distribution on the same line because the active elements are more concentrated, which is not conducive to the mapping of channel knowledge.

**Figure 4:**
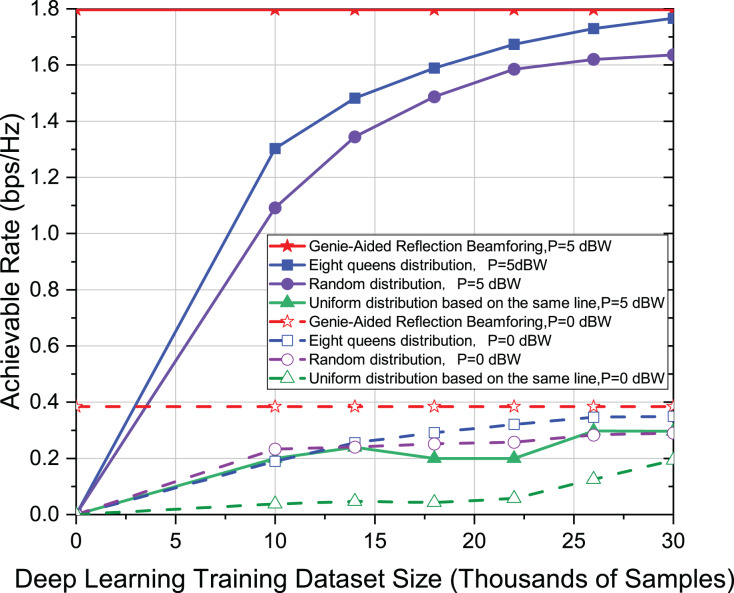
Comparison of achievable rate of eight-queens distribution, random distribution and uniform distribution on the same line when *P* = 5 dBW and *P* = 0 dBW.

To demonstrate the robustness of the proposed eight-queens distribution, several experiments are also conducted in this article. The transmission power adopts *P* = 5 dBW, and *P* = 0 dBW, respectively, in Fig. 4. Other settings are the same as in Fig. 3. The results show that the distribution of eight-queens is still better than other methods when the transmission power is smaller. It can also find the effect of transmission power on the achievable rate. This simulation further demonstrates the robustness of the eight-queens distribution.

**Figure 5:**
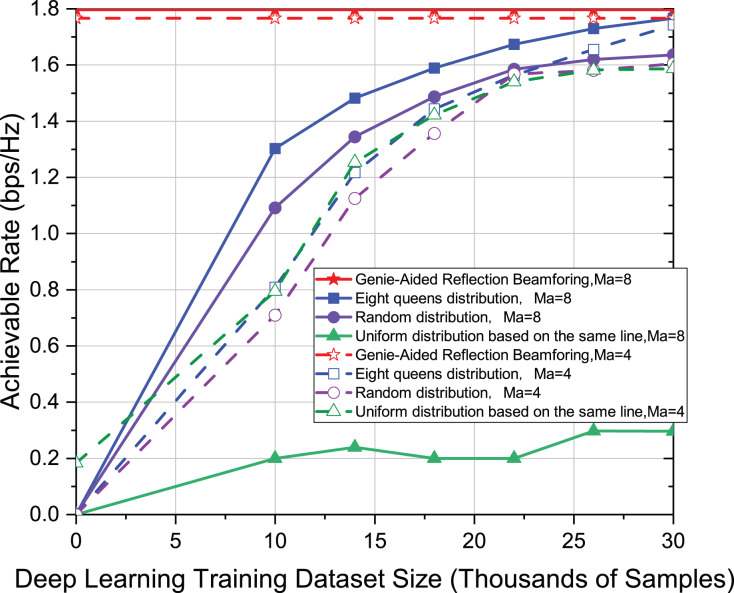
Comparison of achievable rate of eight-queens distribution, random distribution and uniform distribution on the same line when Ma = 8 and Ma = 4.

In Fig. 5, the active elements adopt Ma = 8, and Ma = 4, respectively. The other settings in Fig. 5 are the same as in Fig. 3. We examine the robustness of the eight-queens distribution when the number of active elements varies in Fig. 5. It can be seen from the results that the effect of the eight-queens is still in an optimal position when the number of active elements is four. However, note that both random distribution and uniform distribution on the same line have the same effect in this case. Therefore, it can infer that the influence of the distribution of active elements on the achievable rate is subtle when Ma = 4.

**Figure 6:**
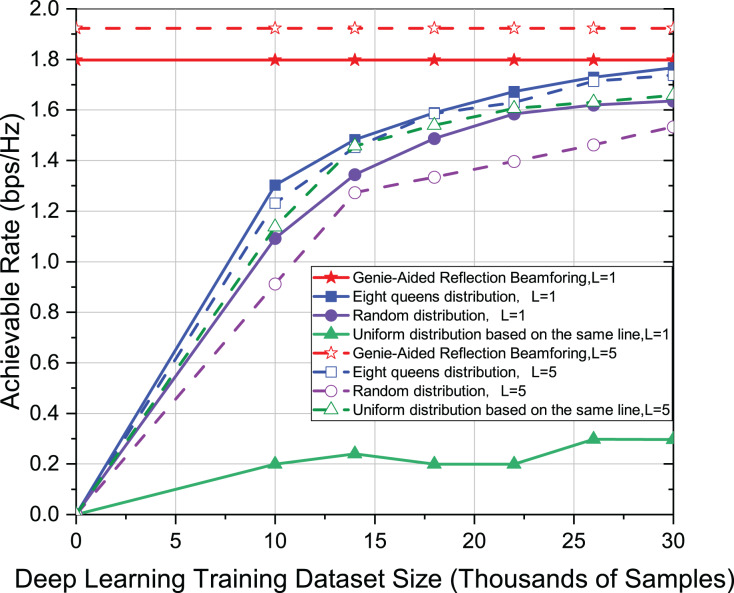
Comparison of achievable rate of eight-queens distribution, random distribution and uniform distribution on the same line when L = 1 and L = 5.

As shown in Fig. 6, when investigating the effect of different distribution patterns of active elements on achievable rates in deep learning models with different dataset sizes, L = 1 and L = 5 are used for channel paths, respectively. Other settings are the same as in Fig. 3. In the case of different channel paths, the eight-queens distribution still has the best advantage. Moreover, when there are more paths, the convergence to the upper limit is slightly slower. For multi-path channels, therefore, the larger data set would be beneficial for model training.

**Figure 7:**
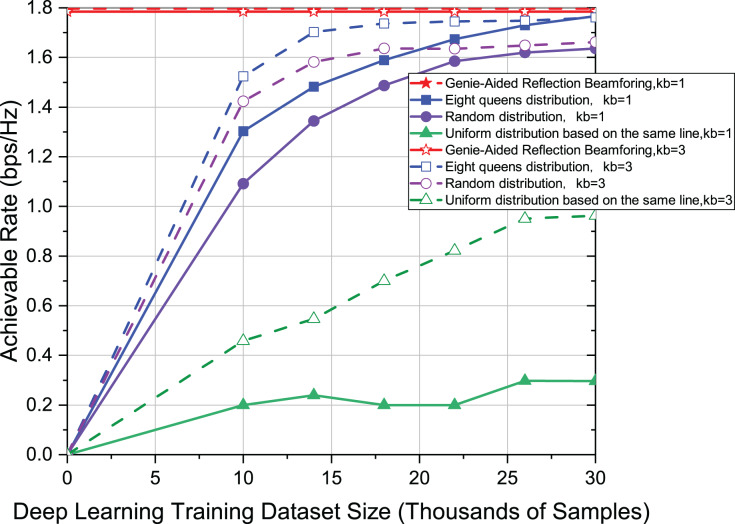
Comparison of achievable rate of eight-queens distribution, random distribution and uniform distribution on the same line when kb = 1 and kb = 3.

It is well known that predicting the kb beams can enhance the reliability of the system. To explore the stability of the eight-queens distribution for different values of kb, Fig. 7 uses kb = 1 and kb = 3, respectively. The other settings in Fig. 7 are the same as in Fig. 3. In Fig. 7, as the value of kb becomes larger, the achievable rate of the system also becomes larger. In particular, the most significant rate improvement can be achieved for distributions in the same line. However, our proposed eight-queens distribution is still the closest to the upper limit. It once again demonstrates the robustness of the novel eight-queens distribution method.

**Figure 8:**
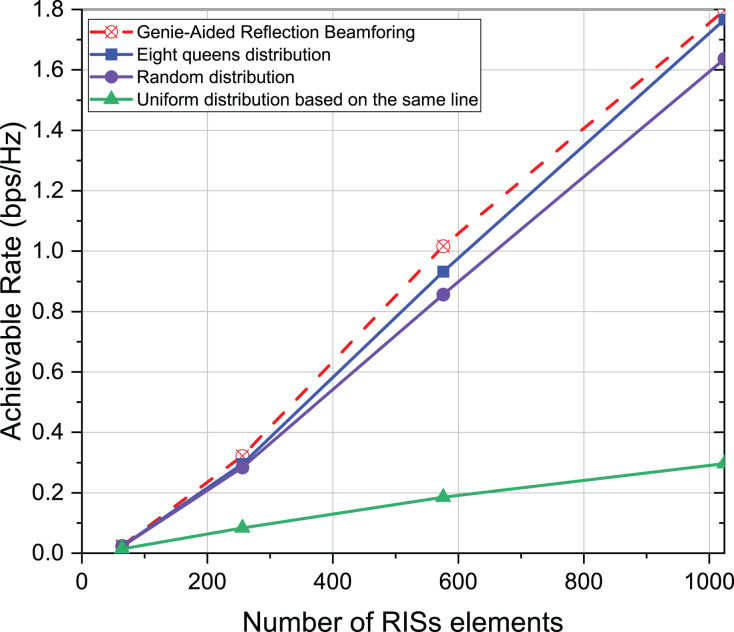
Comparison of achievable rate of eight-queens distribution, random distribution and uniform distribution on the same line when different number of elements.

In addition, the effect of active element distribution is investigated for the achievable rate of the communication system while the number of RISs elements varies. The parameter settings based on Fig. 3 remain unchanged. The number of RISs elements is 64, 256, 576, and 1,024, respectively. According to Fig. 8, the achievable rate of communication system is increasing with the increase of RISs elements. The reason is that the larger the RISs the more channel knowledge is reflected, and the distribution of the eight-queens always has an optimal performance. It proves once again that the distribution of active elements not only affects the achievable rate but the distribution mode of eight-queens is the highest.

## Conculsion

In this article, we analyze the effect of the active elements distribution of the RISs on the achievable rate of the communication system. A novel distribution is proposed: the eight-queens distribution. The random distribution, the uniform distribution on the same line, and the eight-queens distribution, respectively, are compared by simulations. The simulation results show that different distributions of active elements demonstrate different performances, and that the performance of the eight-queens distribution is optimum and is 7% higher than the traditional methods. This is because it guarantees a more decentralized distribution of active elements and a more comprehensive knowledge of the received channel, and that increases the achievable rate of RISs-assisted wireless communication system. Therefore, the distribution structure of RISs active elements should adopt the eight-queens distribution. However, the eight-queens distribution only restricts the active elements not to be in the same row, column, or diagonal. According to the number of active elements and RISs elements, finding the optimal distribution of active elements is still a problem worthy of study. Moreover, the research in this article is carried out based on the assumption of full channel knowledge. Due to the limitation of the hardware and environmental facilities, simulation in a real environment will be our next step.

## Supplemental Information

10.7717/peerj-cs.1207/supp-1 Supplemental Information 1 Source code. This code must be used with the dataset-deepMIMO. The results in the article can be obtained by changing the corresponding parameters in the code.

## Additional Information and Declarations

### Data Availability

The following information was supplied regarding data availability: The deepMIMO data is available at GitHub, https://github.com/lisixu-JLAU/DeepMIMO-V1; lisixu-JLAU. (2022). lisixu-JLAU/DeepMIMO-V1: DeepMIMO-V1 (v1.1.0) [Data set]. Zenodo. https://doi.org/10.5281/zenodo.7101699.
