# Supplementary figures and images for: Achievable rate as affected by active elements distribution in reconfigurable intelligent surfaces for wireless communication
Source: PeerJ Comput Sci. 2023 Jan 12;9:e1207. doi: 10.7717/peerj-cs.1207 (PMC10280233; doi:10.7717/peerj-cs.1207)

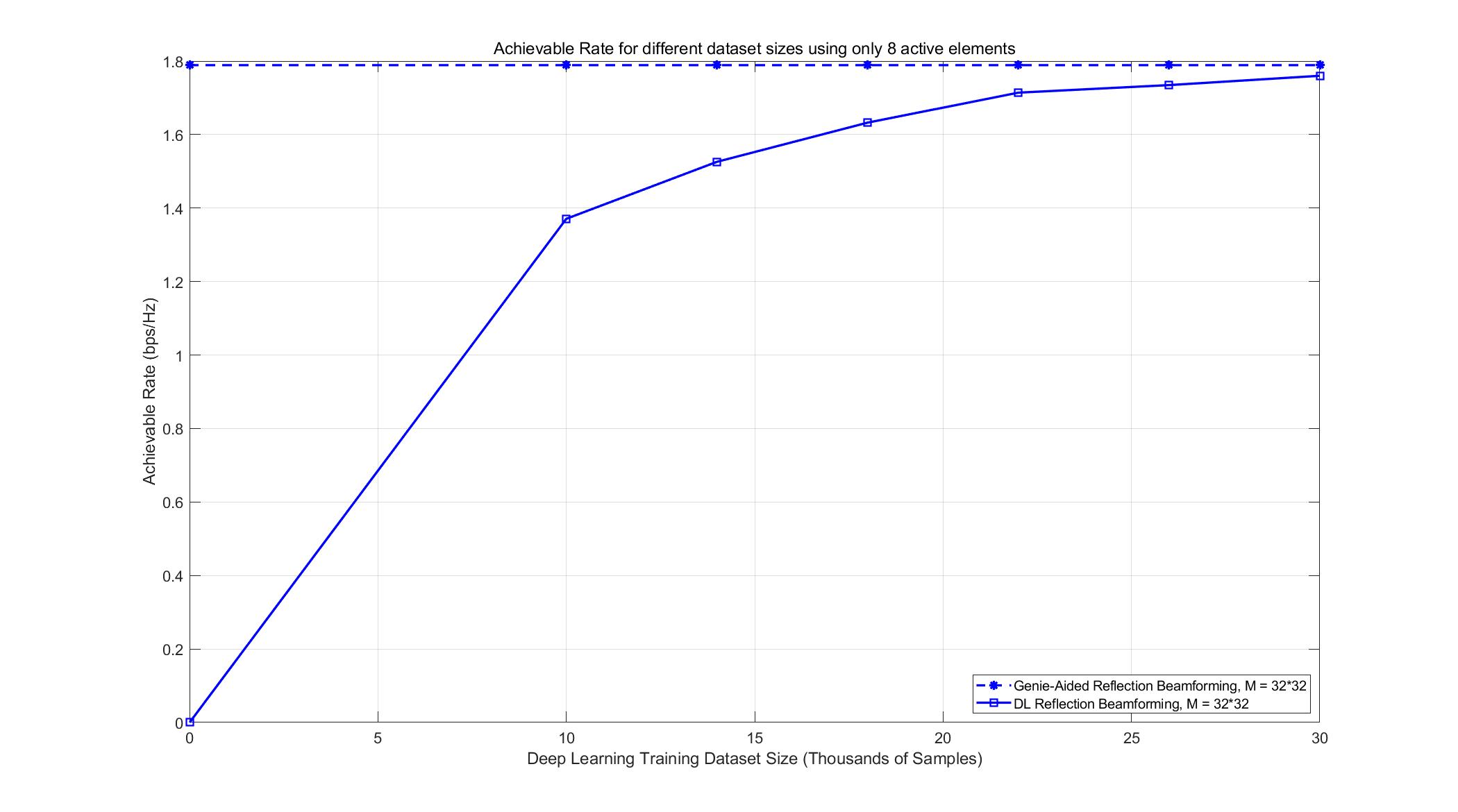

Supplement: Supplemental Information 1 — This code must be used with the dataset-deepMIMO. The results in the article can be obtained by changing the corresponding parameters in the code. [file peerj-cs-09-1207-s001.zip › Source code/202105252106.jpg]

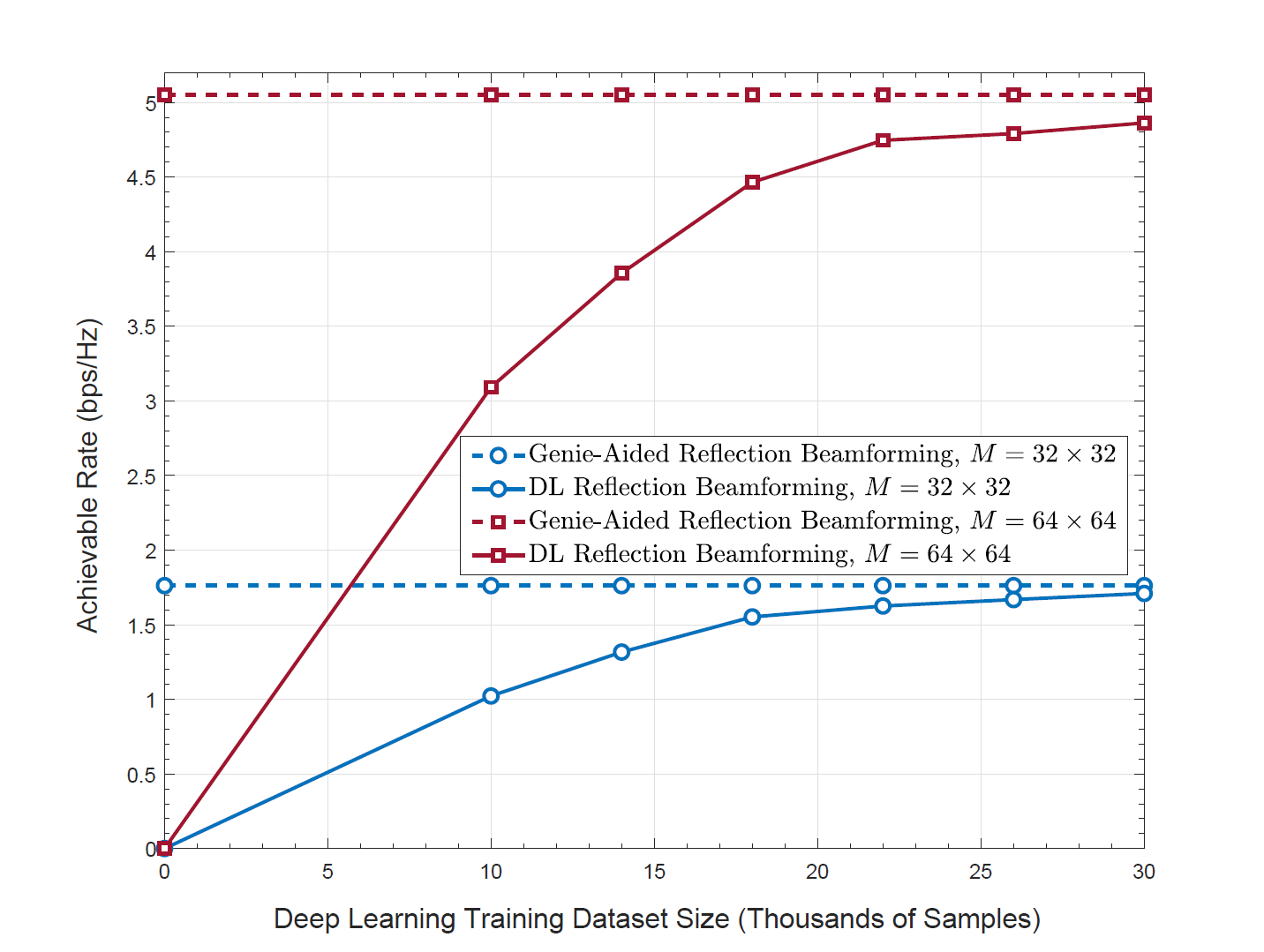

Supplement: Supplemental Information 1 — This code must be used with the dataset-deepMIMO. The results in the article can be obtained by changing the corresponding parameters in the code. [file peerj-cs-09-1207-s001.zip › Source code/Figure12.png]
